# Supplementary figures and images for: Splicing isoforms associated with TGFβ-induced myofibroblast activation
Source: BMC Mol Cell Biol. 2026 Mar 5;27:21. doi: 10.1186/s12860-026-00579-7 (PMC13077938; doi:10.1186/s12860-026-00579-7)

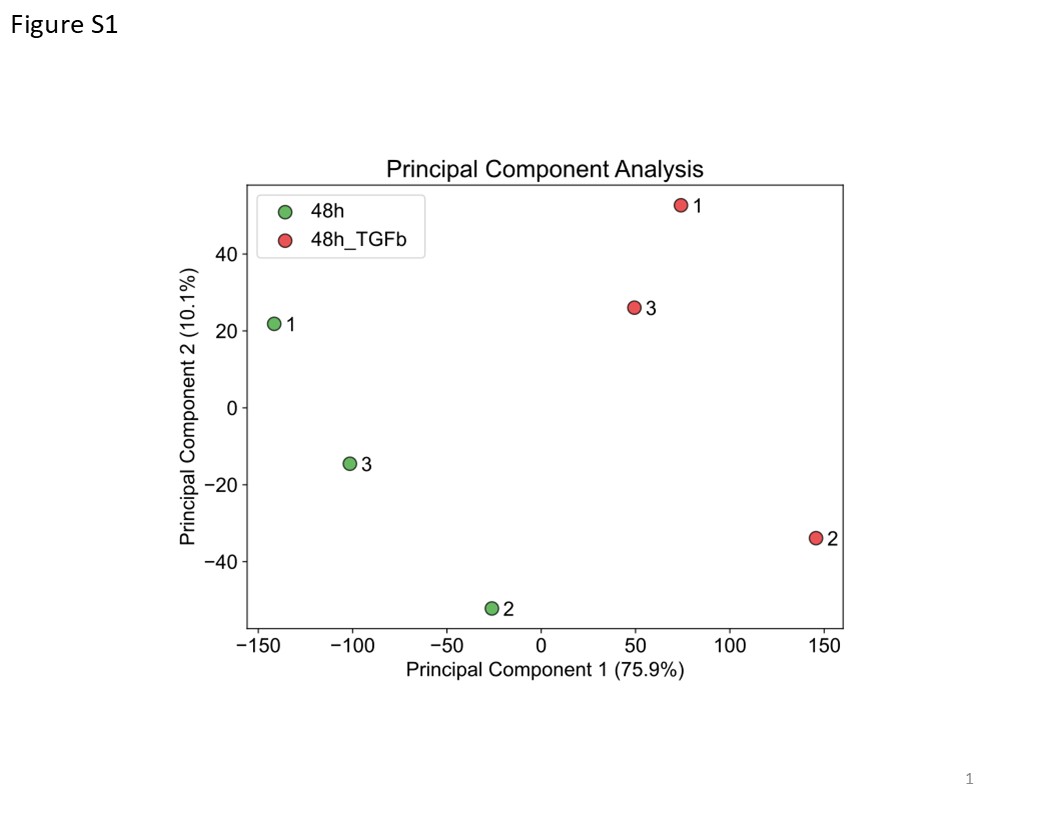

Supplement: Supplementary file 9 — Supplementary Material 9: Figure S1. PCA plot related to the DESeq2 analysis of TGFβ-induced GM05386 fibroblasts [file 12860_2026_579_MOESM9_ESM.jpg]

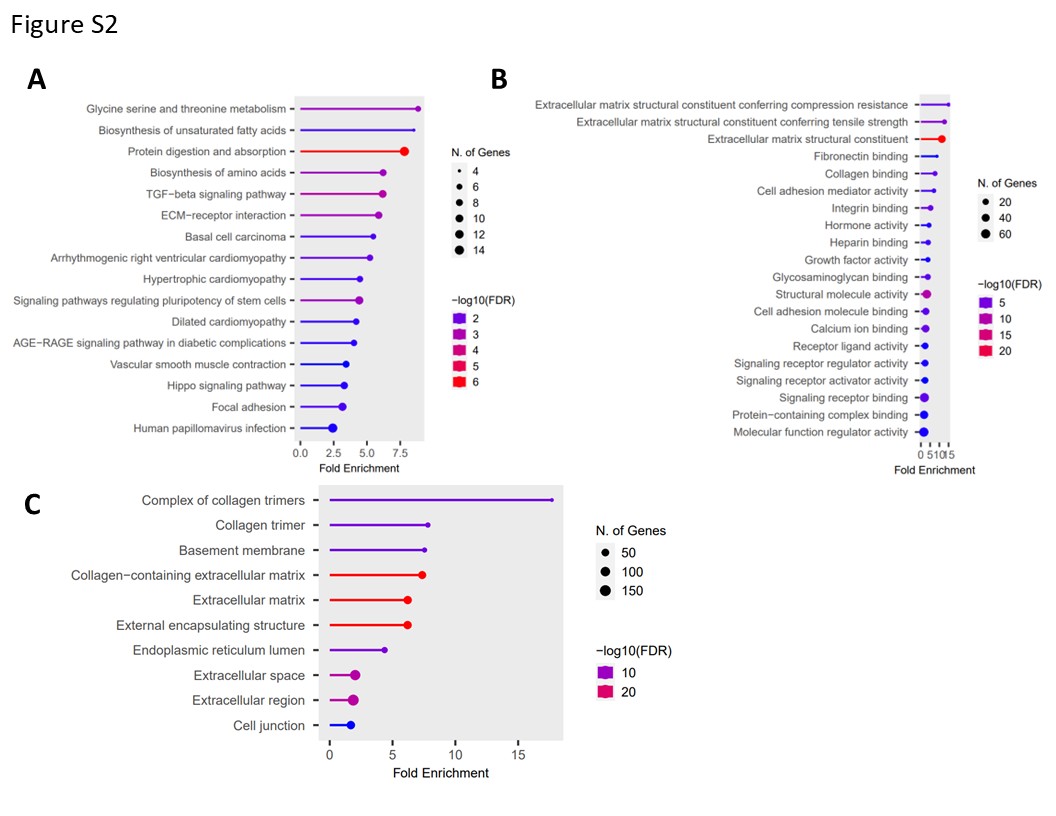

Supplement: Supplementary file 10 — Supplementary Material 10: Figure S2. Gene ontology related to the DESeq2 analysis of TGFβ-induced GM05386 fibroblasts [file 12860_2026_579_MOESM10_ESM.jpg]

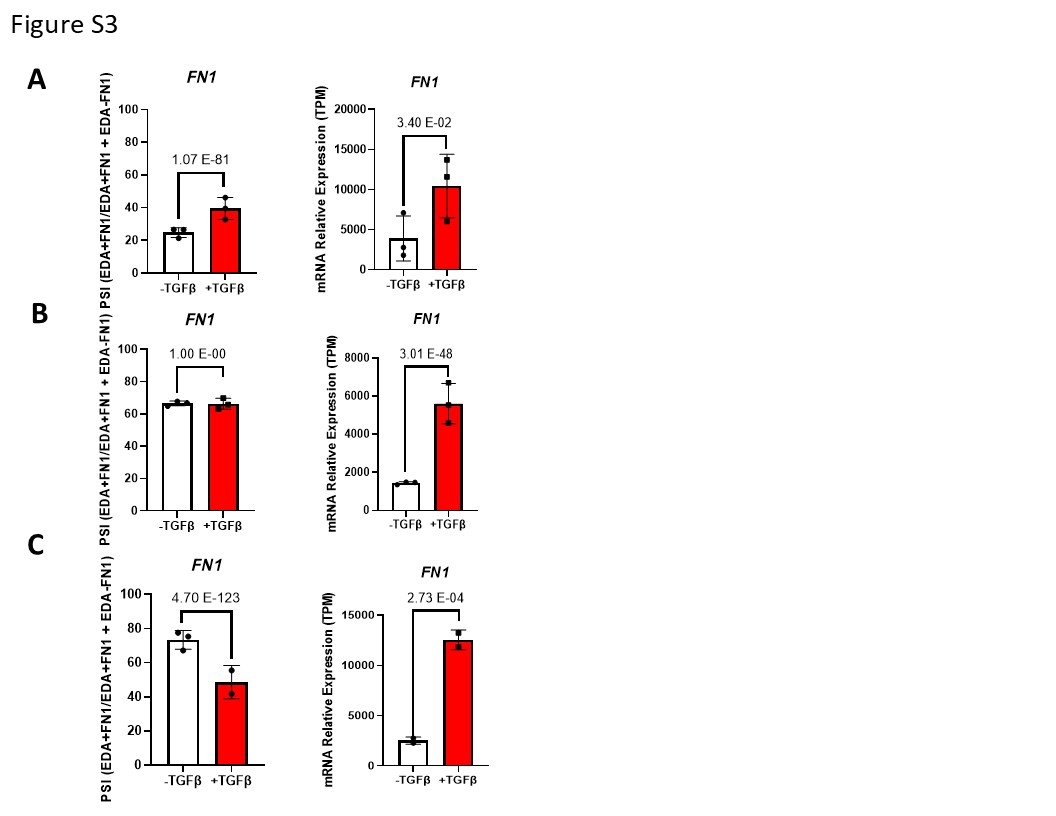

Supplement: Supplementary file 11 — Supplementary Material 11: Figure S3. Fibronectin EDA splicing and global gene expression for, TGFβ-induced fibroblasts from public datasets [file 12860_2026_579_MOESM11_ESM.jpg]
